# Synthesis and Antibacterial Activity of Analogs of 5-Arylidene-3-(4-methylcoumarin-7-yloxyacetylamino)-2-thioxo-1,3-thiazoli-din-4-one

**DOI:** 10.3390/molecules190913577

**Published:** 2014-09-01

**Authors:** Nguyen Tien Cong, Huynh Thi Nhan, Luong Van Hung, Tran Dinh Thang, Ping-Chung Kuo

**Affiliations:** 1 Department of Chemistry, Ho Chi Minh City University of Education, Ho Chi Minh City 70000, Vietnam; E-Mails: congchemist@gmail.com (N.T.C.); nhanhuynhsp@gmail.com (H.T.N.); luonghung365@gmail.com (L.V.H.); 2 Department of Chemistry, Vinh University, Vinh 42000, Vietnam; 3 Department of Biotechnology, National Formosa University, Yunlin 63201, Taiwan

**Keywords:** coumarin, 2-thioxo-1,3-thiazolidin-4-one, spectral elucidation, antibacterial activity

## Abstract

In an effort to develop new antimicrobial agents, 3-(4-methylcoumarin-7-yloxyacetylamino)-2-thioxo-1,3-thiazolidin-4-one (**4**) was synthesized by reaction of thiocarbonylbisthioglycolic acid with ethyl (4-methyl-2-oxo-2*H*-chromen-7-yloxy)aceto- hydrazide (**3**), which was prepared in turn from 7-hydroxy-4-methylcoumarin (**1**). The condensation of compound **4** with different aromatic aldehydes afforded a series of 5-(arylidene)-3-(4-methylcoumarin-7-yloxyacetyl-amino)-2-thioxo-1,3-thiozolidin-4-one analogs **5a**–**h**. The structures of these synthetic compounds were elucidated on the basis of IR, ^1^H-NMR and ^13^C-NMR spectral data and ESI-MS spectrometric analysis. Compounds **5a**–**h** were examined for their antibacterial activity against several strains of Gram-positive and Gram-negative bacteria.

## 1. Introduction

Bacterial disease control, including the food safety issue, has continuously attracted researchers’ attention from various fields. The use of preservatives and pathogen antagonists had been reported as a means of protecting the microbiological safety of fresh and processed food products [1,2,3,4]. Although some antagonists exhibited significant inhibition of bacterial growth, they were too toxic to be utilized long term. In the present study, we hoped to explore new lead compounds with natural skeletons which could be modified for further investigation as antimicrobial agents applied to food preservation. 7-Hydroxy-4-methylcoumarin derivatives with heterocyclic moieties possess diverse biological properties such as antibacterial [5,6,7], antifungal [8,9], anticancer [10], enzyme-inhibitory [11], and antioxidant activities [7,9]. On the other hand, thiazolidin-4-ones are important compounds due to their broad range of biological activities including anticancer [12,13,14], virus-inhibitory [15], HIV-inhibitory [16], and enzyme-inhibitory activities [14]. These observations prompted our interest in synthesizing some new 7-hydroxy-4-methylcoumarin derivatives bearing 2-thioxo-1,3-thiozolidin-4-one substituents and evaluate their antibacterial potential.

## 2. Results and Discussion

### 2.1. Synthesis of 7-Hydroxy-4-methylcoumarin Derivatives ***5a**–**h***

**Scheme 1:**
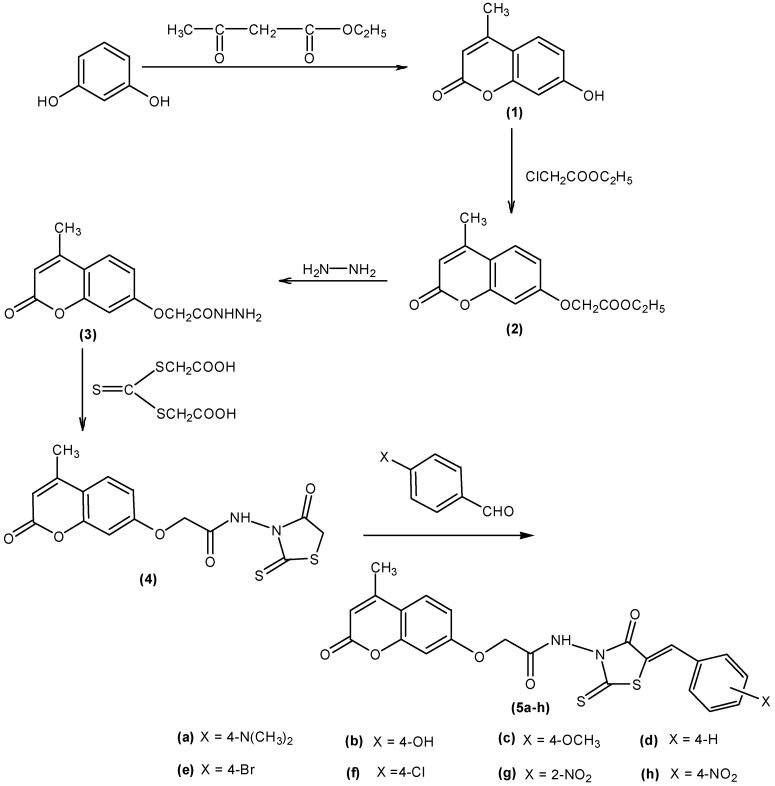
Synthetic route for the preparation of compounds **5a**–**h**.

The synthetic route for the preparation of the target compounds was presented in Scheme 1. Compounds **1**–**3** were synthesized according to the corresponding published procedures [6,7,9,10,11] and characterization of these synthetic compounds was achieved by comparison of their physical and spectral data with those reported in the previous literature [17]. Then compound **3** was reacted with thiocarbonylbisthioglycolic acid in ethanol to obtain new compound **4**, which was converted into the series of *N*-(5-arylidene-4-oxo-2-thioxothiazolidin-3-yl)-2-(4-methyl-2-oxo-2H-chromen-7-yloxy)-acetamides **5a**–**h** by Knoevenagel condensation.

In the IR spectrum of compound **4**, in addition to the lactone and amide carbonyl group absorption at 1709 cm^−1^, the stretching band in a high frequency region (1771 cm^−1^) indicated the presence of C=O bonds in a thiazolidine ring. A new signal with intensity of 2H appearing at 4.47 ppm in the ^1^H-NMR spectrum was attributed to the methylene group of the thiazolidine ring. Another signal also with intensity of 2H appearing in the downfield region at 4.96 ppm was attributed to the oxymethylene protons (OCH_2_). In terms of unexpected results, the signals of the methylene protons in **4** were split instead of being a singlet. The splitting of these signals could be explained by a non-first order splitting effect.

Comparing the ^13^C-NMR spectra of **3** [7] and **4**, three more signals appeared in **4**, two of which were at around 160 ppm corresponding to the signals of the carbon atoms in the thioxo group and carbonyl group, whereas the last one was at 33.4 ppm corresponding to the signal of the saturated carbon atom of the thiazolidine ring. The spectral data of **4** as well as the agreement of the predicted mass with molecular mass determined by HR-MS confirmed that a 4-oxo-2-thioxothiazolidine ring was formed.

In the IR spectrum of compounds **5a**–**h**, there were shifts of the absorption of the carbonyl group at 1771 cm^−1^ to lower frequencies (1721–1755 cm^−1^), in agreement with the formation of a conjugated system between the carbonyl group and the benzylidene moiety. Comparison of the ^1^H-NMR spectra of **5a**–**h** with the ^1^H-NMR of **4** showed not only the disappearance of the methylene proton’s signal at 4.47 ppm, but also appearance of additional aromatic proton signals at 6.84–8.36 ppm and methylidene proton signals at 7.82–8.86 ppm. The signal of the oxymethylene protons (OCH_2_) in compounds **5a**–**h** appeared at 5.00–5.04 ppm. The signals of these protons, like the signals of the methylene protons OCH_2_ in compound **4**, were also split by a non–first order splitting effect. Therefore, they did not appear as singlets.

### 2.2. Determination of the in Vitro Antimicrobial Activity

Compounds **5a**–**h** were examined for antimicrobial activity against *Escherichia coli*, *Pseudomonas aeruginosa* (Gram-negative bacteria), *Bacillus subtilis* and *Staphylococcus aureus* (Gram-positive bacteria) at concentrations of 0.1% and 0.2% according to the reported method with minor modifications [18]. As shown in Table 1, most of the compounds **5a**–**g** at 0.1% exhibited low antimicrobial activity, with antimicrobial inhibition zone diameters of less than 15 mm. However, at the concentration of 0.2%, most of these compounds showed average activity (the antimicrobial diameters were 15 mm to 20 mm) against certain bacteria including *Escherichia coli*, *Pseudomonas aeruginosa* (Gram-negative bacteria), *Bacillus subtilis* and *Staphylococcus aureus* (Gram-positive bacteria). In addition, the synthetic compounds **3** and **4** did not show any significant inhibition of bacterial growth in our preliminary screening and therefore the data were not included.

**Table 1:** Antimicrobial inhibition zone diameters (d) of compounds **5a**–**h**.

Bacteria	Conc.	X							
		4-N(CH_3_)_2_ (5a)	4-OH (5b)	4-OCH_3_ (5c)	4-H (5d)	4-Br (5e)	4-Cl (5f)	2-NO_2_ (5g)	4-NO_2_ (5h)
		d (mm) ^a^							
*Escherichia coli*	0.1%	14	15	13	14	15	12	12	15
	0.2%	16	17	16	16	16	15	15	18
*Pseudomonas aeruginosa*	0.1%	13	19	12	17	15	15	14	18
	0.2%	15	23	15	19	18	17	16	20
*Bacillus subtilis*	0.1%	15	15	14	15	18	14	13	14
	0.2%	17	19	16	18	21	18	16	17
*Staphylococcus aureus*	0.1%	13	14	14	16	17	14	15	16
	0.2%	15	16	18	17	20	16	17	18

^a^ D − d ≥ 25 mm: Very high activity; D − d ≥ 20 mm: High activity; D − d ≥ 15 mm: Average activity; D − d ≤ 15 mm: Low activity. Each experiment was performed in triplicate.

The minimum inhibitory concentration (MIC) value is a measure to define the antibacterial activity of a compound and is defined as the lowest concentration of drug that inhibits visible growth. Compounds **5a**–**h** were subjected to examination of their MIC values according to the reported method [19] and the data are shown in Table 2. The two-fold microdilution broth method was used and all of the tested samples demonstrated inhibitory effects in a concentration-dependent manner. However, only **5c**, **5g** and **5h** exhibited any significant inhibition against *S. aureus* with MIC values of 50 μg/mL.

**Table 2:** The minimum inhibitory concentrations (MICs) of **5a**–**h** against bacteria.

Bacteria	MIC (μg/mL)							
	4-N(CH_3_)_2_ (5a)	4-OH (5b)	4-OCH_3_ (5c)	4-H (5d)	4-Br (5e)	4-Cl (5f)	2-NO_2_ (5g)	4-NO_2_ (5h)
*Escherichia coli*	- ^a^	-	-	-	-	-	-	-
*P. aeruginosa*	-	-	-	-	-	-	-	-
*Bacillus subtilis*	-	-	-	-	-	-	-	-
*Staphylococcus aureus*	-	-	50	-	-	-	50	50

^a^ MIC > 50 μg/mL and not determined.

## 3. Experimental Section

### 3.1. General Procedures

All starting materials were purchased from Merck (Darmstadt, Germany) and used without purification. Melting points were measured in open capillary tubes on a Gallenkamp melting point apparatus. The structures of all compounds were confirmed by IR, NMR and HR-MS spectra. IR spectra were recorded on a Shimadzu FTIR-8400S spectrometer using KBr pellets. The ^1^H-NMR spectra were recorded on a Bruker Avance spectrometer at 500 MHz using DMSO-d_6_ as solvent, while the ^13^C-NMR, HSQC, HMBC spectra were recorded at 125 MHz. The data are given in parts per million (ppm) and are referenced to an internal standard of tetramethylsilane (TMS, δ 0.00 ppm). The spin-spin coupling constants (*J*) are given in Hz. Peak multiplicities are reported as *s* (singlet), *d* (doublet), *dd* (double-doublet), *t* (triplet), *q* (quartet), and *m* (multiplet). The MS spectra were recorded on a Bruker microTOF-Q 10187 spectrometer or on a Varian FT-ICR-MS 910 spectrometer.

### 3.2. Synthesis of 7-Hydroxy-4-methylcoumarin Derivatives ***5a**–**h***

#### 3.2.1. Synthesis of 4-Oxo-2-thioxothiazolidine Derivatives

Compounds **1**–**3** were prepared using the corresponding reported methods [6,7,9,10,11] as shown in Scheme 1.

#### 3.2.2. Synthesis of 2-(4-Methyl-2-oxo-2*H*-chromen-7-yloxy)-*N*-(4-oxo-2-thioxothiazo-lidin-3-yl)aceta-mide (**4**)

A mixture of (4-methyl-2-oxo-2*H*-chromen-7-yloxy)acetohydrazide (**3**, 0.01 mol) and thio-carbonylbisthioglycolic acid (0.01 mol) in ethanol (5 mL) was refluxed for 8 h. After cooling the resulting solid was filtered off, dried and recrystallized from HOAc/DMF to give compound **4** as a yellowish powder in 74.0% yield; mp: 248–249 °C; IR (ν, cm^−1^): 3258, 3100, 2901, 1771, 1709, 1622, 1491, 1425, 1391, 1358, 1298, 1245; ^1^H-NMR (ppm) δ 11.41 (1H, *s*, NH), 7.71 (1H, *d*, ^3^*J* = 9.0 Hz, H-5), 7.04 (1H, *dd*, ^3^*J* = 9.0 Hz, ^4^*J* = 2.5 Hz, H-6), 7.01 (1H, *d*, ^4^*J* = 2.5 Hz, H-8), 6.23 (1H, *s*, H-3), 4.96 (2H, OCH_2_), 4.47 (2H, CH_2__thiazolidine ring_), 2.39 (3H, *s*, CH_3_); ^13^C-NMR (ppm) δ 199.7 (C=S), 170.1 (NH–CO–CH_2_), 165.8 (>N–CO), 160.3 (C-7), 160.0 (O–C=O), 154.4 (C-9), 153.3 (C-4), 126.5 (C-5), 112.5 (C-6), 113.8 (C-10), 111.6 (C-3), 101.8 (C-8), 66.0 (OCH_2_), 33.4 (SCH_2_), 18.1 (CH_3_); HR-ESI-MS *m/z* 365.0266 [M+H]^+^ (calcd. for C_15_H_13_N_2_O_5_S_2_, 365.0266). 

#### 3.2.3. General Procedure for Synthesis of *N*-(5-Arylidene-4-oxo-2-thioxothiazolidin-3-yl)-2-(4-methyl-2-oxo-2H-chromen-7-yloxy)acetamides **5a**–**h**

Equimolar amounts of **4** (5.0 mmol), anhydrous sodium acetate (5.0 mmol) and an appropriate aromatic aldehyde (5.0 mmol) in glacial acetic acid (5 mL) were refluxed for 5 h. The reaction mixture was cooled and the solid separated was filtered and recrystallized to give compounds **5a**–**h**.

*N-{5-[4-(Dimethylamino)benzylidene]-4-oxo-2-thioxothiazolidin-3-yl}-2-(4-methyl-2-oxo-2H-chromen-7-yloxy)acetamide* (**5a**): Red powder; Yield: 69.0; mp. 265–266 °C; IR (ν, cm^−1^): 3314, 3088, 2915, 1721, 1616, 1574, 1530, 1441, 1385, 1302, 1263; ^1^H-NMR (δ, ppm): 3.06 (6H, *s*, –N(CH_3_)_2_), 2.41 (3H, *s*, CH_3_), 5.00 (2H, OCH_2_); 6.25 (1H, *s*, 3-H), 7,05 (1H, *d*, ^4^*J* = 2.5, 8-H), 7.07 (1H, *dd*, ^3^*J* = 9.0, ^4^*J* = 2.5, 6-H), 7.72 (1H, *d*, ^3^*J* = 8.5, 5-H), δ 7.79 (1H, *s*, =CH-), 11.6 (1H, *s*, NH), 7.50 (2H, *d*, ^3^*J* = 9.0) and 6.84 (2H, *d*, ^3^*J* =9.0) (H_benzene ring_); ^13^C-NMR (δ, ppm): 160.0 (O–C=O), 111.6 (3-C), 153.3 (4-C), 18.1 (CH_3_), 126.5 (5-C), 112.6 (6-C), 160.3 (7-C), 101.9 (8-C), 154.5 (9-C), 113.9 (10-C), 66.1 (OCH_2_), 166.0 (–NH–CO–CH_2_), 163.2 (>N–CO), 113.9 (>C= _thiazolidine ring_), 189.6 (C=S), 136.3 (=CH–), 119.6, 133.6, 112.6 and 112.3 (C_benzene ring_), 152.2 (C_arom_–N(CH_3_)_2_), 39.6 (–N(CH_3_)_2_); HR-ESI-MS: 496.1001 (M+H), calcd. for (C_24_H_21_N_3_O_5_S_2_): 495.0923. 

*N-(5-(4-Hydroxybenzylidene)-4-oxo-2-thioxothiazolidin-3-yl)-2-(4-methyl-2-oxo-2H-chromen-7-yloxy)acetamide* (**5b**): Brown yellow powder; yield: 64%; mp: 284–285 °C; IR (ν, cm^−1^): 3340, 2920, 2851, 1732, 1709, 1678, 1572, 1510, 1483, 1397, 1364, 1242, 1229; ^1^H-NMR (δ, ppm): 10.70 (1H, *s*, OH), 2.41 (3H, *s*, CH_3_), 5.01 (2H, OCH_2_), 6.25 (1H, *s*, 3-H), 7.05 (1H, *d*, ^4^*J* = 2.5, 8-H), 7.06 (1H, *dd*, ^3^*J* = 9.0, ^4^*J* = 2.5, 6-H), 7.73 (1H, *d*, ^3^*J* = 8.5, 5-H), 7.86 (1H, *s*, =CH-), 11.7 (1H, *s*, NH), 7.58 (2H, *d*, ^3^*J* = 9.0) and 6.95 (2H, *d*, ^3^*J* = 9.0) (H_benzene ring_); ^13^C-NMR (δ, ppm): 160.0 (O–C=O), 111.6 (3-C), 153.4 (4-C), 18.1 (CH_3_), 126.5 (5-C), 112.7 (6-C), 160.3 (7-C), 101.9 (8-C), 154.5 (9-C), 113.9 (10-C), 66.1 (OCH_2_), 166.1 (–NH–CO–CH_2_), 163.2 (>N–CO), 114.3 (>C= _thiazolidine ring_), 190.1 (C=S), 135.7 (=CH–), 123.8, 133.7 and 116.7 (C_benzene ring_), 161.2 (C_arom_–OH); HR-ESI-MS: 469.0497 (M+H), calcd. for (C_22_H_16_N_2_O_6_S_2_): 468.0450.

***N****-(5-(4-Methoxybenzylidene)-4-oxo-2-thioxothiazolidin-3-yl)-2-(4-methyl-2-oxo-2H-chromen-7-yloxy)acetamide* (**5c**): Yellow powder; yield: 71.0%; mp: 239–240 °C; IR (ν, cm^−1^): 3205, 3080, 2936, 1736, 1699, 1618, 1510, 1479, 1389, 1314, 1258, 1229, 1180, 1134; ^1^H-NMR (δ, ppm): 6.26 (1H, *d*, ^4^*J* = 1.0, 3-H), 2.41 (3H, *d*, ^4^*J* = 1.0, CH_3_), 7.72 (1H, ^3^*J* = 8.5, 5-H), 7.08 (1H, *dd*, ^3^*J* = 8.5; ^4^*J* = 2.5, 6-H), 7.05 (1H, *d*, ^4^*J* = 2.5, 8-H), 5.02 (2H, OCH_2_), 11.70 (1H, *s*, NH), 7.92 (1H, *s*, =CH-), 7.68 (2H, *d*, ^3^*J* = 8.5) and 7.15 (2H, *d*, ^3^*J* = 8.5) (H_benzene ring_), 3.85 (3H, *s*, OCH_3_); ^13^C-NMR (δ, ppm): 160.0 (O–C=O), 111.6 (3-C), 153.3 (4-C), 18.1 (CH_3_), 126.5 (5-C), 112.6 (6-C), 160.3 (7-C), 101.9 (8-C), 154.5 (9-C), 113.9 (10-C), 66.1 (OCH_2_), 166.0 (–NH–CO–CH_2_), 163.2 (>N–CO), 115.6 (>C= _thiazolidine ring_), 190.0 (C=S), 135.2 (=CH–), 125.2, 133.3 and 115.2 (C_benzene ring_), 161.9 (C_arom_–OCH_3_), 55.6 (OCH_3_); HR-ESI-MS: 505.0494 (M+Na), calcd. for (C_23_H_18_N_2_O_6_S_2_): 482.0606.

***N****-(5-Benzylidene-4-oxo-2-thioxothiazolidin-3-yl)-2-(4-methyl-2-oxo-2H-chromen-7-yloxy)acetamide* (**5d**): Yellow powder; yield: 54.0%; mp: 249–250 °C; IR (ν, cm^−1^): 3376, 3080, 1740, 1719, 1622, 1564, 1479, 1427, 1370, 1296, 1256, 1220; ^1^H-NMR (δ, ppm): 2.41 (3H, *s*, CH_3_), 5.02 (2H, OCH_2_); 6.25 (1H, *s*, 3-H), 7.05 (1H, *d*, ^4^*J* = 2.5, 8-H), 7.08 (1H, *dd*, ^3^*J* = 8.5, ^4^*J* = 2.0, 6-H), 7.74 (1H, *d*, ^3^*J* = 8.5, 5-H), 7.96 (1H, *s*, =CH-), 7.69 (2H, *d*, ^3^*J* =7.0) and 7.57 (3H, *m*) (H_benzene ring_); ^13^C-NMR (δ, ppm): 160.0 (O–C=O), 111.6 (3-C), 153.3 (4-C), 18.1 (CH_3_), 126.5 (5-C), 112.6 (6-C), δ 160.3 (7-C), 101.9 (8-C), 154.5 (9-C), 113.9 (10-C), 66.1 (OCH_2_), 166.1 (–NH–CO–CH_2_), 163.1 (>N–CO), 119.8 (>C= _thiazolidine ring_), 190.1 (C=S), 135.1 (–CH=), 132.7, 130.9, 131.4 and 129.6 (C_benzene ring_); HR-ESI-MS: 453.0478 (M+H), calcd. for (C_22_H_16_N_2_O_5_S_2_): 452.0501.

***N****-(5-(4-Bromobenzylidene)-4-oxo-2-thioxothiazolidin-3-yl)-2-(4-methyl-2-oxo-2H-chromen-7-yloxy)acetamide* (**5e**): Yellow powder; yield: 73.0%; mp: 279–280 °C; IR (ν, cm^−1^): 3214, 3100, 1736, 1697, 1618, 1605, 1578, 1487, 1440, 1387, 1269, 1258, 1240; ^1^H-NMR (δ, ppm): 2.41 (3H, *s*, CH_3_), 5.02 (2H, OCH_2_); 6.30 (1H, *s*, 3-H), 7.04 (1H, *d*, ^4^*J* = 2.5, 8-H), 7.06 (1H, *dd*, ^3^*J* = 8.5, ^4^*J* = 2.5, 6-H), 7.74 (1H, *d*, ^3^*J* = 8.5, 5-H), 7.97 (1H, *s*, =CH–), 7.76 (2H, *d*, ^3^*J* = 9.0) and 7.62 (2H, *d*, ^3^*J* = 9.0) (H_benzene ring_); ^13^C-NMR (δ, ppm): 160.0 (O–C=O), 111.6 (3-C), 153.3 (4-C), 18.1 (CH_3_), 126.5 (5-C), 112.6 (6-C), 160.3 (7-C), 101.8 (8-C), 154.5 (9-C), 113.9 (10-C), 66.1 (OCH_2_), 166.1 (–NH–CO–CH_2_), 163.0 (>N–CO), 119.9 (>C= _thiazolidine ring_), 189.8 (C=S), 133.8 (=CH–),131.8 and 132.6 (C_benzene ring_), 125.1 (C_arom_–Br); HR-ESI-MS: 530.9671 (M+H) and 532.9651 [M+H+2], calcd. for (C_22_H_15_BrN_2_O_5_S_2_): 529.9606 and 531.9606.

***N****-(5-(4-Chlorobenzylidene)-4-oxo-2-thioxothiazolidin-3-yl)-2-(4-methyl-2-oxo-2H-chromen-7-yloxy)acetamide* (**5f**): Yellow powder; yield: 70.0%; mp: 275–276 °C; IR (ν, cm^−1^): 3215, 3084, 2,934, 1736, 1697, 1605, 1584, 1489, 1476, 1441, 1387, 1370, 1269, 1258, 1242, 1132; ^1^H-NMR (δ, ppm): 6.30 (1H, *s*, 3-H), 2.41 (3H, *s*, CH_3_), 7.74 (1H, *d*, ^3^*J* = 8.5; 5-H), 7.07 (1H, *dd*, ^3^*J* = 8.5, ^4^*J* = 2.5, 6-H), 7.05 (1H, *d*, ^4^*J* = 2.5, 8-H), 5.02 (2H, OCH_2_), 11.7 (1H, *s*, NH), 7.97 (1H, *s*, =CH–), 7.72 (2H, *d*, ^3^*J* = 8.5) and 7.64 (2H, *d*, ^3^*J* = 8.5) (H_benzene ring_); ^13^C-NMR (δ, ppm): 160.0 (O–C=O), 111.6 (3-C), 153.3 (4-C), 18.1 (CH_3_), 126.5 (5-C), 112.6 (6-C), 160.3 (7-C), 101.8 (8-C), 154.5 (9-C), 113.9 (10-C), 66.0 (OCH_2_), 166.1 (–NH–CO–CH_2_), 163.0 (>N–CO), 119.8 (>C= _thiazolidine ring_), 189.8 (C=S), 136.1 (=CH–), 131.5, 132.5 and 129.6 (C_benzene ring_), 133.7 (C_arom_–Cl); HR-ESI-MS: 509.0004 (M+Na), calcd. for (C_22_H_15_ClN_2_O_5_S_2_): 486.0111.

*2-(4-Methyl-2-oxo-2H-chromen-7-yloxy)-N-(5-(2-nitrobenzylidene)-4-oxo-2-thioxothiazolidin-3-yl)acetamide* (**5g**): Pale pink powder; yield: 57.0%; mp: 260–261 °C; IR (ν, cm^−1^): 3316, 3080, 2940, 1755, 1717, 1616, 1522, 1483, 1387, 1343, 1298, 1271; ^1^H-NMR (δ, ppm): 6.30 (1H, *s*, 3-H), 2.41 (3H, *s*, CH_3_), 7.74 (1H, *d*, ^3^*J* = 8.5, 5-H), 7.07 (1H, *dd*, ^3^*J* = 8.5, ^4^*J* = 2.5, 6-H), 7.05 (1H, *d*, ^4^*J* = 2.5, 8-H), 5.03 (2H, OCH_2_), 11.6 (1H, *s*, NH), 8.22 (1H, *s*, =CH–), 7.81 (1H, *d*, ^3^*J* = 7.5), 7.79 (1H, *dd*, ^3^*J*_1_ = ^3^*J*_2_ = 7.5), 7.91 (1H, *dd*, ^3^*J*_1_ = ^3^*J*_2_= 7.5) and 8.26 (1H, *d*, ^3^*J* = 7.5) (H_benzene ring_); ^13^C-NMR (δ, ppm): 160.0 (O–C=O), 111.6 (3-C), 153.3 (4-C), 18.1 (CH_3_), 126.6 (5-C), δ 112.6 (6-C), 160.3 (7-C), 101.9 (8-C), 154.5 (9-C), 113.9 (10-C), 66.0 (OCH_2_), 166.1 (–NH–CO–CH_2_), 162.3 (>N–CO), 123.4 (>C= _thiazolidine ring_), 190.3 (C=S), 134.8 (=CH–),147.8 (C_arom_–NO_2_), 131.7, 125.7, 129.6, 132.3 and 128.3 (C_benzene ring_); HR-ESI-MS: 498.04295 (M+H), calcd. for (C_22_H_15_N_3_O_7_S_2_): 497.0351.

*2-(4-Methyl-2-oxo-2H-chromen-7-yloxy)-N-(5-(4-nitrobenzylidene)-4-oxo-2-thioxothiazolidin-3-yl)acetamide* (**5h**): Yellow powder; yield: 60%; mp: 273–274 °C; IR (ν, cm^−1^): 3235, 3084, 2940, 1736, 1697, 1609, 1526, 1474, 1442, 1387, 1346, 1269, 1238, 1200; ^1^H-NMR (δ, ppm): 6.30 (1H, *s*, 3-H), 2.41 (3H, *s*, CH_3_), 7.73 (1H, *d*, ^3^*J* = 9.0, 5-H), 7.07 (1H, *dd*, ^3^*J* = 9.0, ^4^*J*= 2.5, 6-H), 7.04 (1H, *d*, ^4^*J* = 2.5, 8-H), 5.04 (2H, OCH_2_), 11.7 (1H, *s*, NH), 8.08 (1H, *s*, =CH–), 8.36 (2H, *d*, ^3^*J* = 8.5) and 7.95 (2H, *d*, ^3^*J* = 8.5) (H_benzene ring_); ^13^C-NMR (δ, ppm): 160.0 (O–C=O), 111.6 (3-C), 153.3 (4-C), 18.1 (CH_3_), 126.5 (5-C), 112.6 (6-C), 160.3 (7-C), 101.8 (8-C), 154.5 (9-C), 113.9 (10-C), 66.0 (OCH_2_), 166.1 (–NH–CO–CH_2_), 162.9 (>N–CO), 123.4 (>C= _thiazolidine ring_), 189.6 (C=S), 132.2 (=CH–), 147.9 (C_arom_–NO_2_), 138.7, 124.4 and 131.7 (C_benzene ring_); HR-ESI-MS: 520.0239 (M+Na), calcd. for (C_22_H_15_N_3_O_7_S_2_): 497.0351.

### 3.3. Determination of the in Vitro Antimicrobial Activity

The compounds **5a**–**h** at concentrations of 0.1% and 0.2% were examined for antimicrobial activity against *Escherichia coli* (ATCC 25922), *Pseudomonas aeruginosa* (ATCC 25923) (Gram-negative bacteria), *Bacillus subtilis* (ATCC 11774) and *Staphylococcus aureus* (ATCC 11632) (Gram-positive bacteria) according to the reported method with minor modifications [18]. A mixture of meat extract (5.0 g), peptone (5.0 g), NaCl (5.0 g), agar (20.0 g) and distilled water (1000 mL) was stirred to dissolve the ingredients and then sterilized in an autoclave to give the MPA environment for growing the bacteria. The mixture was poured to Petri dishes. The Petri dishes then were put in a sterile cabinet for 24 h. After infusion of the particular bacteria into the MPA environment in the Petri dishes, a hole was drilled in the center of the dish. DMSO solution (0.1 mL) of the particular chemical at a concentration of 0.1% or 0.2% was dripped into the hole. The samples were placed in a refrigerator for 4–8 h, and then incubated at room temperature for 24 h. The inhibiting zone was measured by the (D − d) value expressed in millimeters (mm), where D was the diameter of inhibited zone and d was the diameter of the hole. The evaluation was based on the following criteria: D − d ≥ 25 mm: very strong antibacterial activity; D − d ≥ 20 mm: strong antibacterial activity; D − d ≥ 15 mm: medium antibacterial activity; D − d ≤ 15 mm: weak antibacterial activity. Each experiment was performed in triplicate.

### 3.4. Minimum Inhibitory Concentration (MIC) Determination

The amount of growth in the wells containing test samples was compared with the amount of growth in the control wells when determining the growth end points. When a single skipped well occurred, the highest MIC was read. Each experiment was performed in triplicate. Streptomycin and tetracyclin were used as positive controls for Gram-positive bacteria and Gram-negative bacteria, respectively.

## 4. Conclusions

Eight new 5-arylidene-3-(4-methylcoumarin-7-yloxyacetylamino)-2-thioxo-1,3-thiozolidin-4-one analogs **5a**–**h** were successfully synthesized. The structures of these compounds were determined by IR, ^1^H-NMR, ^13^C-NMR and HR-ESI-MS spectral data. Most of the compounds **5a**–**h** exhibited significant activity against *Bacillus subtilis* and *Staphylococcus aureus*, *Escherichia coli* and *Pseudomonas aeruginosa* at a concentration of 0.2%*.* Further structural modification could be performed to improve the bioactivity and may prove useful in developing new therapeutic anti-microbial agents.
